# Analysis of sex-differential gene expression on the target of approved drug

**DOI:** 10.1038/s41598-025-12342-7

**Published:** 2025-07-24

**Authors:** Yunha Suh, Jun-Gyu Lee, Kwang-eun Kim

**Affiliations:** 1 Organelle Medicine Research Center, Yonsei University Wonju College of Medicine, Wonju, 26426 Republic of Korea; 2 Department of Convergence Medicine, Yonsei University Wonju College of Medicine, Wonju, 26426 Republic of Korea; 3 Department of Global Medical Science, Yonsei University Wonju College of Medicine, Wonju, 26426 Republic of Korea

**Keywords:** Drug safety, Health policy, Gene expression

## Abstract

Sex is a key piece of patient information but is often not actively considered in drug use. This is partly due to the lack of molecular evidence at the gene expression level beyond sex chromosomes and sex hormones. We aim to investigate how sex differences in tissue-specific gene expression relate to FDA**-**approved drugs using the latest database of The Genotype-Tissue Expression (GTEx) V10. Our analysis reveals that 91.4% of FDA-approved drug target genes exhibit sex-differential expression in at least one tissue. The tissues with the most pronounced sex differences include subcutaneous adipose tissue, skeletal muscle, and the pituitary gland, while sex differences are less pronounced in the liver, other brain regions, and the spleen. Sex-differential disease-related genes include those associated with obesity (PPARG, INSR), cancer (FGFR1, CD22), and immunity (IL6R, IL3RA). Based on our findings, we advocate for a policy shift that integrates sex-based molecular data into preclinical studies, drug development, and clinical practices. This paradigm aligns biomedical research with precision medicine, mitigates drug-related risks, and promotes equitable healthcare outcomes.

## Introduction

Sex is undeniably a fundamental biological variable. It is widely recognized that children are not small adults, emphasizing the fundamental physiological differences between children and adults^1^. Similarly, sex differences go beyond simple distinctions like height and weight; women are not small men. In a study of 13,000 US adults, the average height and weight of men were 176.2 cm and 86.9 kg, respectively, compared to 162.1 cm and 74.2 kg for women. Notably, the average body fat percentage for men was 28.1%, while for women, it was 40.0%, highlighting significant differences in lipid metabolism between the sexes^2^. The genetic differences between men and women are estimated to be around 1–2%, which may influence disease development, progression, and treatment^3,4^.

For example, autoimmune diseases exemplify sex-based differences in disease prevalence^5^. Alzheimer’s disease (AD) also exhibits significant sex differences. The X-linked ubiquitin-specific peptidase 11 (USP11) is more highly expressed in women and is thought to promote tau protein aggregation through tau deubiquitination^6^. Moreover, research on sex differences is actively being conducted across various types of cancer^7–11^. These findings underscore the critical role of sex as a biological variable in both disease mechanisms and therapeutic responses.

Historically, therapeutics aimed to treat all individuals equally using a one-size-fits-all approach. However, personalized strategies, known as precision medicine, are becoming increasingly essential. Sex, as a fundamental biological variable, serves as a starting point for precision medicine through sex-specific strategies. While significant progress has been made in emphasizing the importance of sex differences, more research is needed to fully understand how these differences impact disease and medicine. Additionally, social and cultural factors may influence gene expression.

This highlights the potential for individuals to respond differently to the same medication. For example, zolpidem, a widely prescribed drug for insomnia, was found to cause morning driving impairments in women due to slower clearance rates, even after adjusting for body weight. As a result, in 2013, the US Food and Drug Administration (FDA) reduced the recommended dose for women by half^12^. Furthermore, several studies have reported sex-related differences in both the efficacy and adverse effects of medications targeting the central nervous system or psychotropic drugs^13,14^.

Reflecting this understanding, the FDA’s Office of Women’s Health has stated, ‘Biological differences may contribute to variations in the safety and efficacy of medical products.’ The FDA has reported that women experience adverse drug reactions more frequently than men, based on data from the Adverse Event Reporting System (AERS). 46% of drugs (307 out of 668) in the US have demonstrated sex-based differences in adverse drug events, with women reporting a greater number of unique drug-event combinations than men. Research conducted in South Korea has also shown that adverse effects associated with antidiabetic medications are more frequently observed in female patients^15,16^.

Even though the National Institutes of Health (NIH) has mandated the consideration of sex and race in funded clinical trials since 1993^17^, a review of 107 randomized controlled trials that included both men and women found that 72% did not incorporate sex as a variable in their analyses^18^. Similarly, pharmaceutical companies often exclude pre-approval data on sex differences in their New Drug Applications (NDAs), and the FDA has been reported to have failed to fully adhere to its own regulations in the drug approval process^19^.

Sex differences are often overlooked in biomedical research. An analysis of cancer cell line reports revealed that only 36.5% of studies specified the sex of the cells^20^. A 2011 study found that research on male animals was 5.5 times more common than research on female animals^21^, primarily due to concerns that hormonal fluctuations associated with the female reproductive cycle could introduce variability into experimental results. However, comprehensive assessments of hundreds of behavioral indicators have demonstrated that female rodents do not exhibit greater overall behavioral variability than males^22–24^. There is a growing need to consider and report sex differences more extensively in biomedical research^25,26^.

Sex differences evident in animal and cell models. For instance, research on stem cells has shown that female muscle-derived stem cells regenerate skeletal muscle in mouse models more efficiently than male cells^27^. This underscores the importance of incorporating sex differences not only in clinical studies involving humans but also in biomedical research. In response to these findings, the US National Institutes of Health has announced mandatory sex-specific analysis in 2014^28^, and Horizon Europe has encouraged research that accounts for sex and gender. More recently, the MESSAGE (Medical Science Sex and Gender Equity) project was launched in the United Kingdom. Recent studies have shown that research funding and journal editorial policies that consider sex/gender differences have contributed to improvements in sex/gender research^29^.

While much research has focused on sex hormones and sex chromosomes in relation to sex differences, our understanding at the broader genetic level remains limited. For instance, the CYP19A1 gene, which encodes aromatase, a key enzyme that converts testosterone to estradiol, is located on chromosome 15. This highlights that sex differences cannot be fully explained by sex chromosomes or hormones alone, underscoring the importance of studying tissue-specific global gene expression differences.

The Genotype-Tissue Expression (GTEx) project was established to investigate the genetic effects on the transcriptome across human tissues and link these findings to traits and disease associations. The project collected samples from 54 non-diseased tissues of 981 donors, and the gene expression data for each tissue are publicly accessible through the GTEx Portal. A previous study using V8 of the dataset reported that 37.5% of the human transcriptome exhibits sex-specific differential expression in at least one tissue^30^. Studies continue to report the presence of sex differences in gene expression across tissues in various datasets^31–33^. Building on this research, we aimed to emphasize its clinical significance and applied the same methodology to analyze FDA-approved drug target genes using the latest GTEx V10, released in November 2024.

Our study differs from previous publications in three key aspects. First, earlier studies utilized older versions of the database, such as GTEx V6^33^ or V8^30,31^. Compared to V8, GTEx V10 includes approximately 12% more RNA-Seq data. For instance, the number of samples for Adipose—Subcutaneous increased from 663 in V8 to 714 in V10. Second, while prior studies focused primarily on biological aspects, they lacked detailed discussion regarding drug development, efficacy, and usage in the therapeutic context. Our study aims to address this gap by emphasizing clinically relevant interpretations. Third, when limiting the analysis to FDA-approved drug target genes, we observed notable differences in the ranking of tissues showing sex-biased gene expression. These discrepancies likely arise from the increased sample size and our focus on drug-target genes.

## Results

**Fig. 1:**
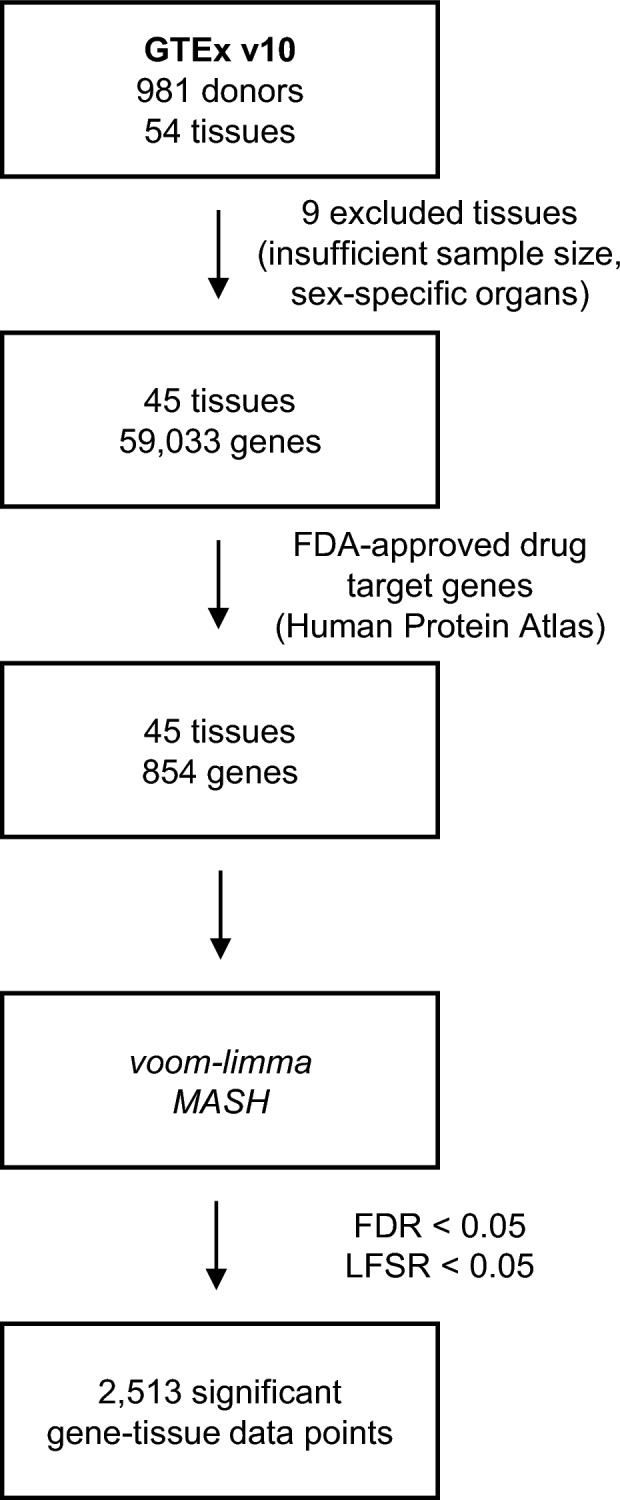
Analysis workflow of sex-differential FDA-approved drug target genes**.** False Discovery Rate, FDR < 0.05, Local False Sign Rate, LFSR < 0.05.

We analyzed 45 out of 54 non-diseased tissues (excluding 1 tissue with insufficient sample size and 8 sex-specific tissues), along with 854 FDA-approved drug target genes provided by the Human Protein Atlas. Using the same statistical methods as in a previous study^30^, we identified significant differences in 6.5% (2513/38,430) of the possible tissue-gene combinations (Fig. 1).

**Fig. 2:**
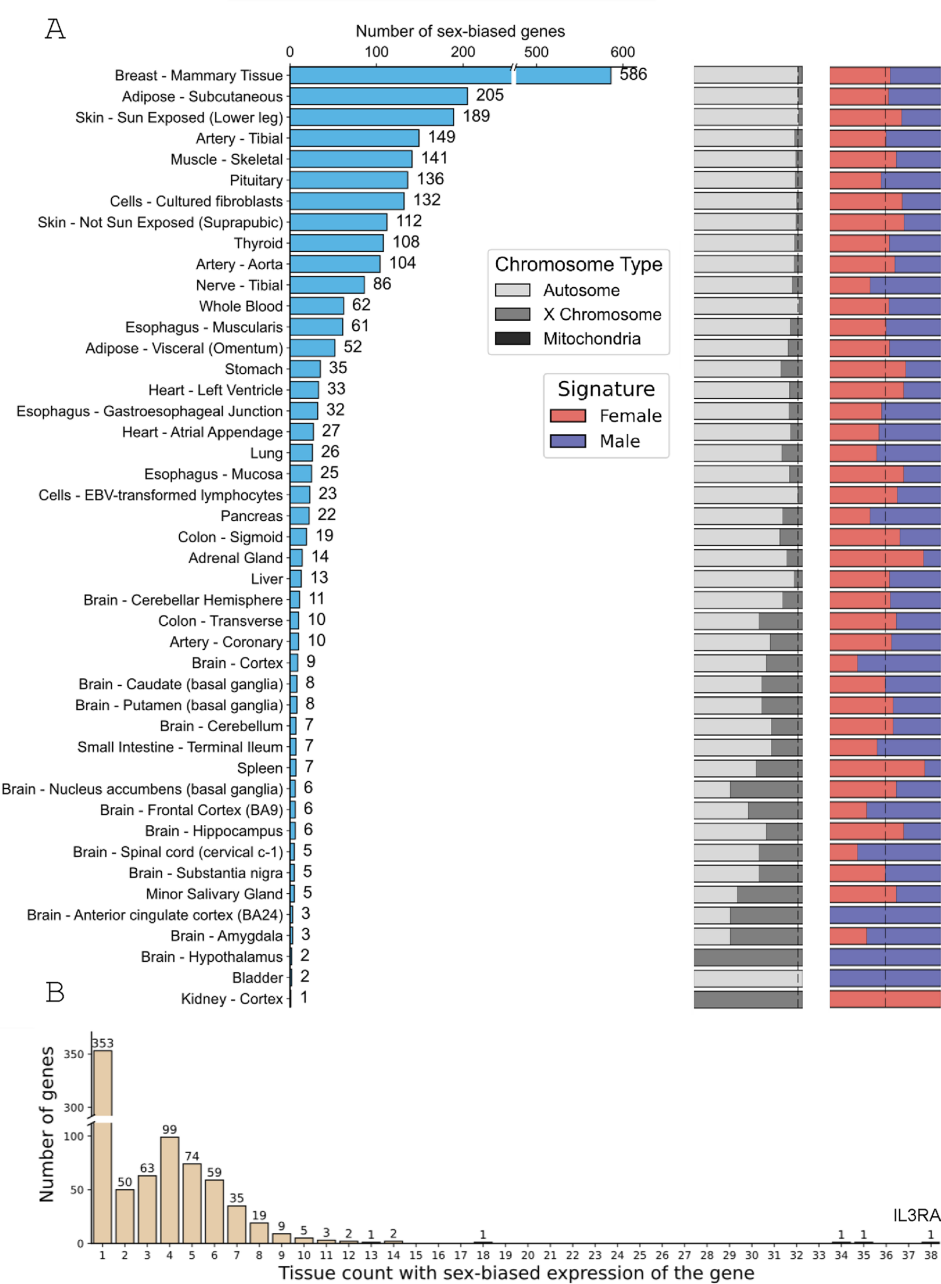
Number of sex-differential FDA-approved drug target genes. (**A**) A histogram illustrates the number of sex-biased genes in each tissue, while stacked bar plots show the proportions of X-linked versus autosomal sex-biased genes, as well as female-versus male-biased genes. Dashed vertical lines are provided as a reference, representing the expected proportion. (**B**) A histogram illustrates the distribution of sex-biased gene expression across tissues. The x-axis represents the number of tissues in which each gene exhibits significant sex-biased expression, and the y-axis indicates the number of genes corresponding to each count of significantly sex-biased tissues.

As expected, breast tissue exhibited the largest sex differences in gene expression. Interestingly, pronounced differences were also noted in subcutaneous adipose tissue, skeletal muscle, and pituitary, while liver, other brain regions, and spleen exhibited relatively minimal differences (Fig. 2A, Supplementary Data 1). Most genes with sex differences were located on autosomes, followed by the X chromosomes. Among mitochondrial genes, only MT-ND1 (NADH ubiquinone oxidoreductase chain 1) showed significant differences in breast tissue. Next, we analyzed whether there are genes that show sex differences across multiple tissues. As a result, 91.1% of the genes (778/854) exhibited sex differences in at least one tissue, and 49.5% of the genes (425/854) showed differences in at least two tisseus. Notably, IL3RA (Interleukin 3 receptor, alpha also called CD123) displayed sex-biased differences in up to 38 tissues (Fig. 2B, Supplementary Data 1).

**Fig. 3:**
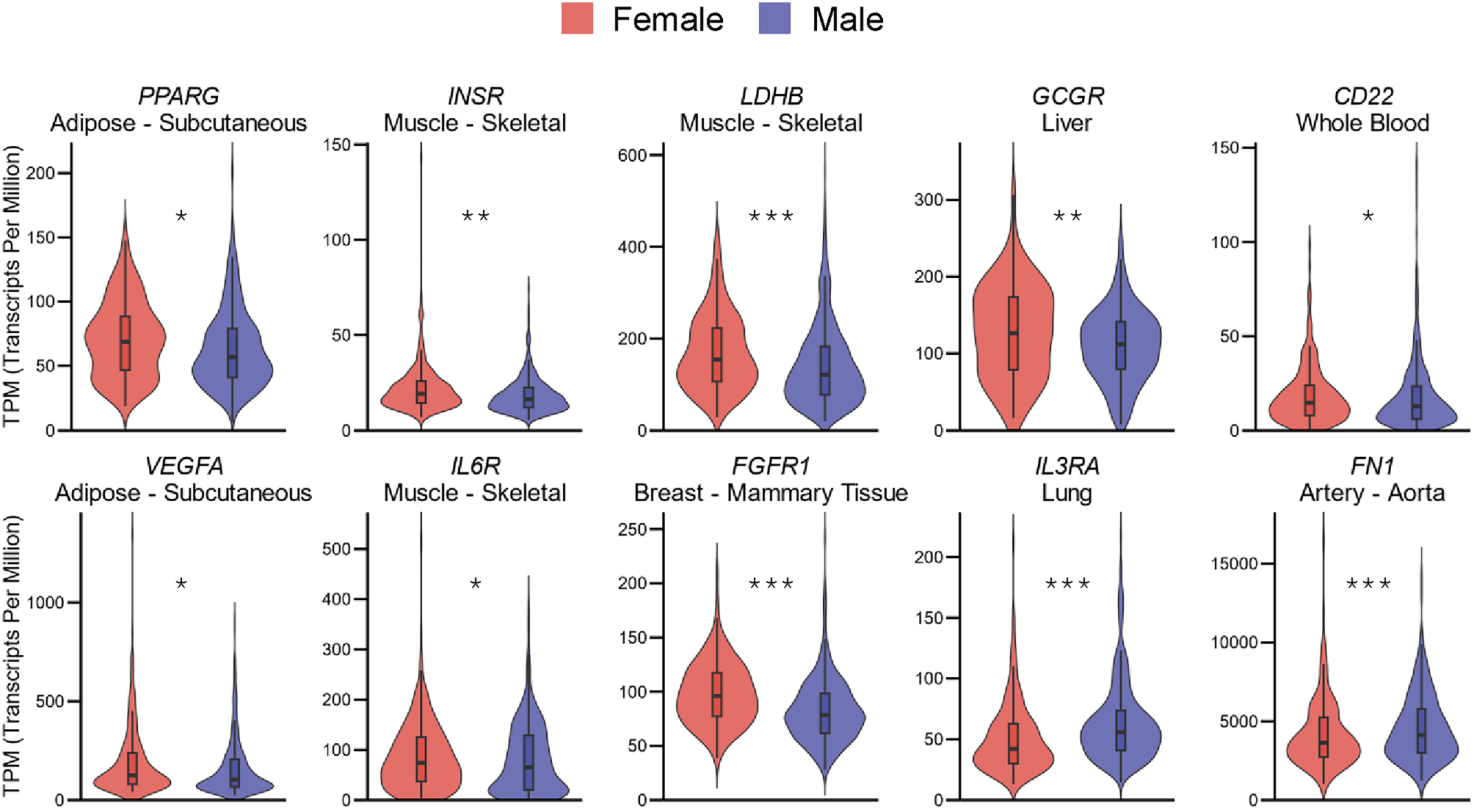
Representative genes with significant sex differences. Selected example of significant genes. *P < 0.05, **P < 0.005, ***P < 0.001. PPARG, peroxisome proliferator activated receptor gamma; VEGFA, vascular endothelial growth factor A; INSR, insulin receptor; IL6R, interleukin 6 receptor subunit alpha; LDHB, L-lactate dehydrogenase B chain; FGFR1, fibroblast growth factor receptor 1; GCGR, glucagon receptor; IL3RA, interleukin-3 receptor subunit alpha; CD22, B-cell receptor CD22; FN1, fibronectin.

We identified several notable genes. In subcutaneous adipose tissue, PPARG (peroxisome proliferator activated receptor gamma) and VEGFA (vascular endothelial growth factor A) showed differential expression, while INSR (insulin receptor), IL6R (interleukin 6 receptor subunit alpha), and LDHB (L-lactate dehydrogenase B chain) exhibited sex differences in skeletal muscle (Fig. 3).

PPARG acts as a key nuclear transcription factor in subcutaneous adipose tissue, regulating adipocyte differentiation and insulin sensitivity^34^. VEGFA in subcutaneous adipose tissue promotes angiogenesis and maintains proper blood supply, supporting metabolic homeostasis of the tissue^35^. INSR mediates glucose uptake and metabolism in skeletal muscle, playing a central role in regulating blood glucose levels^36^. IL6R interacts with IL-6 secreted by skeletal muscle during stimuli such as exercise, modulating inflammation and energy metabolism^37^. LDHB is involved in lactate metabolism and the glycolysis/gluconeogenesis pathway in skeletal muscle, contributing significantly to muscle energy regulation^38^.

Other genes that showed significant differences include FGFR1 (fibroblast growth factor receptor 1) in the breast, GCGR (glucagon receptor) in the liver, IL3RA (interleukin-3 receptor subunit alpha) in the lungs, CD22 (B-cell receptor CD22) in whole blood, and FN1 (fibronectin) in the aorta (Fig. 3).

FGFR1 in breast tissue (particularly in breast cancer) drives cell proliferation, survival, and angiogenesis, thus influencing tumor growth and progression^39^. GCGR in the liver is crucial for glucose homeostasis by regulating hepatic glucose output in response to glucagon signaling^40^. IL3RA regulates the proliferation and differentiation of bone marrow progenitor cells^41^. CD22, predominantly expressed on B lymphocytes, modulates antigen receptor signaling and helps regulate B-cell activation and immune responses^42^. FN1 contributes to the structural integrity of the aortic vessel wall and facilitates smooth muscle cell adhesion, playing a key role in vascular elasticity and function^43^.

**Fig. 4:**
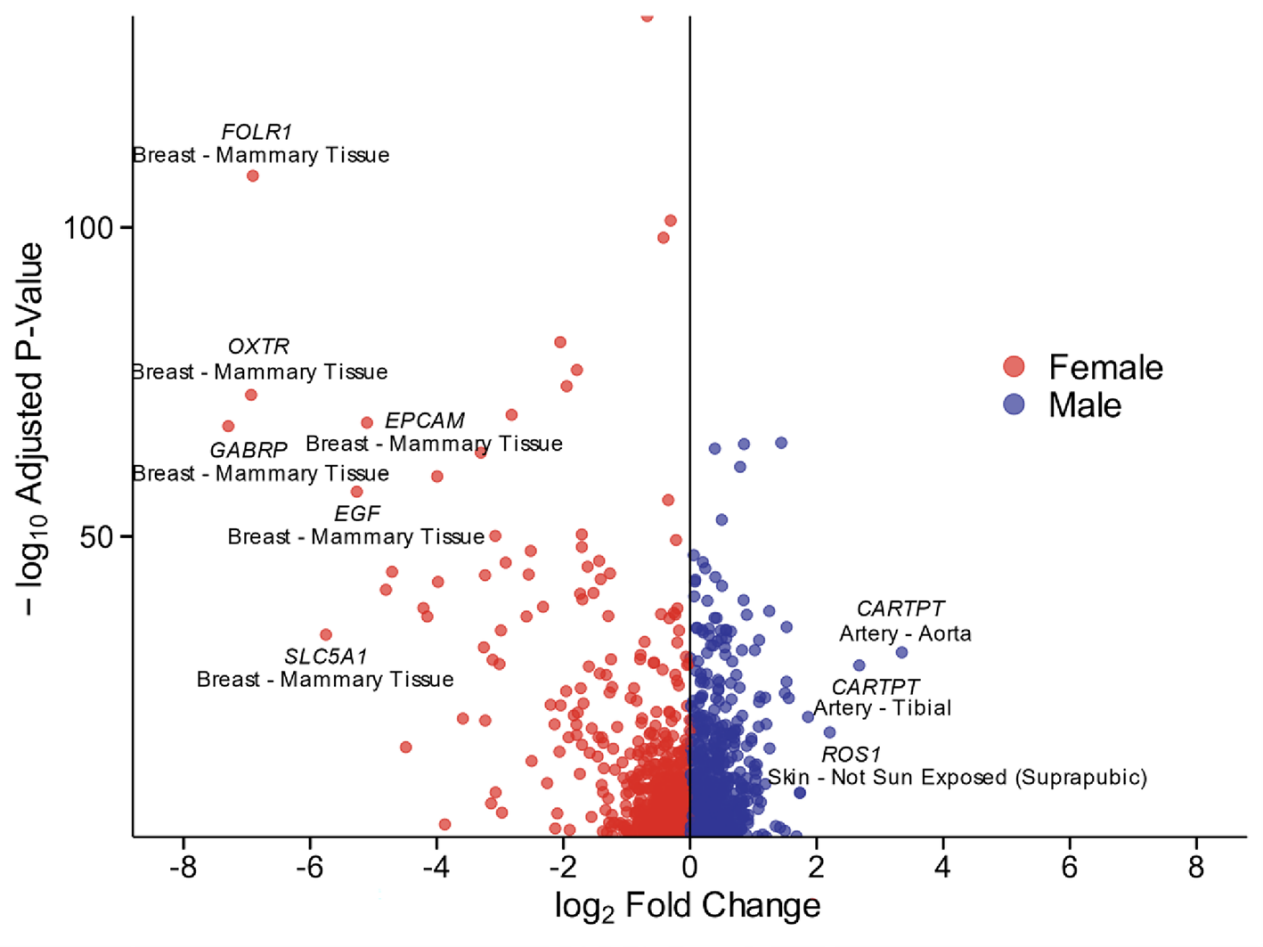
Volcano plot depicting significant sex-differential genes. Adjusted *P*-value < 0.05, LFSR < 0.05. GABRP, gamma-aminobutyric receptor subunit pi; FOLR1, folate receptor alpha; OXTR, oxytocin receptor; SLC5A1, sodium/glucose cotransporter 1; EGF, pro-epidermal growth factor; EPCAM, epithelial cell adhesion molecule; CARTPT, cocaine- and amphetamine-regulated transcript protein; ROS1, proto-oncogene tyrosine-protein kinase ROS.

When comparing median expression differences, more genes were expressed at higher levels in females than in males, with the majority of these genes detected in breast tissue (Fig. 4, Supplementary Data 1). Genes that were showed higher expression in females included GABRP (gamma-aminobutyric receptor subunit pi), FOLR1 (folate receptor alpha), OXTR (oxytocin receptor), SLC5A1 (sodium/glucose cotransporter 1, also called SGLT1), EGF (pro-epidermal growth factor), and EPCAM (epithelial cell adhesion molecule).

GABPR is known to regulate mitochondrial biogenesis, cell growth, and gene expression^44^. FOLR1 in breast cancer cells facilitates cellular uptake of folate and can enhance tumor proliferation, making it a potential target for cancer therapy^45^. OXTR mediates oxytocin signaling in the mammary glands, promoting milk ejection and lactation post-partum^46^. SLC5A1 actively transports glucose to maintain energy and glucose homeostasis^47^. EGF regulates proliferation and differentiation of breast epithelial cells and plays a pivotal role in tumor cell growth in breast cancer^48^. EPCAM, expressed on the surface of breast cancer cells, is involved in cell adhesion and signaling, serving as a useful marker for cancer diagnosis and targeted therapy^49^.

Conversely, genes with higher expression in males included CARTPT (cocaine- and amphetamine-regulated transcript protein) in the arteries and ROS1 (proto-oncogene tyrosine-protein kinase ROS) in the skin. CARTPT regulates appetite and energy balance^50^. ROS1 can drive tumor cell growth and survival through its oncogenic fusion proteins^51^.

## Discussion

In this study, we analyzed sex differences in the expression of 854 FDA-approved drug target genes across 45 non-diseased tissues, discovering that over 90% of these genes exhibited sex-biased expression in at least one tissue. Significant differences were most evident in breast, subcutaneous adipose tissue, skeletal muscle, and pituitary. Key therapeutic target genes, such as PPARG (targeted by pioglitazone and rosiglitazone), VEGFA (targeted by bevacizumab and ranibinumab), IL6R (target by satralizumab and tocilizumab) and CD22 (target by inotuzumab ozogamicin and moxetumomab pasudotox), exhibited notable sex differences in their expression. We suggest that investigating the relationship between drug effects or toxicity and genes with sex-biased expression across tissues could offer valuable insights. For example, while sex differences in adverse event reports at the system organ class level have been studied, the role of gene expression in these effects remains unexplored^15,16^. Future studies could propose optimal dosages or combination therapies that enhance efficacy and reduce toxicity based on sex differences.

Some genes from our analysis have evidence of sex differences in human or mouse studies experiments. In examining the OXTR rs53576 variant, Tost et al.^52^ found that males carrying the A (risk) allele showed more pronounced alterations in brain structure and social behavior than females. This study sheds light on possible biological underpinnings of male-biased susceptibility to certain social or developmental conditions. Pparg mRNA was decreased in the gonadal white adipose tissue (gWAT) of young C57BL/6 J male mice fed a high-fat diet, whereas it was increased in the gWAT of young female mice^53^. Vegfa mRNA levels were higher in gWAT of FVB;B6 female mice than male mice, however, this difference was reversed under a high-fat diet^54^. Both SLC5A1 mRNA and protein levels were higher in females in brush border membrane isolated from the kidney of adult Wistar strain rats^55^. EGF concentrations measured in serum and submandibular glands were higher in adult male Swiss-Webster mice^56^.

If sex differences are considered during the basic research and preclinical stages of drug development, it would be possible to develop safer and more effective drugs^57,58^. While mice are predominantly used in basic research, most studies are conducted exclusively on male mice. By referencing GTEx V10 data, preclinical animal experiments could be designed to better reflect human sex differences. Additionally, sex-specific pharmacokinetic and pharmacodynamic information could help predict potential adverse effects^59^. This information could also facilitate drug repurposing or the expansion of indications, reducing pharmaceutical companies’ development costs and risks.

The historical practice of excluding women of reproductive age from clinical trials has resulted in a lack of sex-specific data, and efforts to address this gap have been reported^60^. A review of 50 years of data shows that adverse drug reactions are 1.5 times more likely to be reported in women than in men^61^. Additionally, the U.S. Government Accountability Office (GAO) found that eight out of ten drugs withdrawn from the market between 1997 and 2001 posed a higher risk of adverse effects in women^62^. Therefore, it is crucial to ensure balanced female participation in clinical trials. By categorizing participants by sex and adjusting dosing protocols accordingly, the success rate of clinical trials could be improved. This approach could minimize healthcare costs by enabling more effective treatments and reducing related adverse reactions.

Even after a drug has been marketed, its efficacy and side effects may vary according to sex, so careful monitoring is required during post-market surveillance. There is growing evidence that considering sex differences may lead to more discoveries^63^. By systematically reanalyzing cancer risk by sex and adiposity, researchers could propose safer and more effective therapeutic interventions^64^. Collecting real world data to build real world evidence is crucial, and if systematic reviews and meta-analyses on drug responses by sex are published, they could lead to the establishment of guidelines or standards.

While we have emphasized RNA sequencing-based gene expression methods, there are some limitations. The GTEx V10 dataset includes only healthy individuals, meaning expression changes may differ under disease conditions. Additionally, gene expression patterns can vary between humans and mice. It is also important to recognize that the biological functions are ultimately carried out by proteins. In this regard, future research should expand to incorporate proteomic techniques, such as mass spectrometry, proximity extension assays, and spatial proteomics, all of which hold significant potential in the field of pathology.

Through these holistic approaches, we can gain a deeper understanding of the relationship between biological sex, a fundamental trait since the dawn of humanity, and diseases such as obesity, cancer, and the pandemics that the world faces in the present era.

## Methods

The GTEx v10 dataset includes 19,616 RNA-seq samples collected from 981 post-mortem donors, comprising 654 males and 327 females. Among these, 18,293 samples (from 77-bladder to 818-skeletal muscle) from 45 tissues were used for analysis after excluding 9 tissues due to insufficient sample size (Kindey—Medulla) or their association with sex-specific organs (e.g., Prostate, Testis, Ovary, Uterus, Cervix—Ectocervix, Cervix—Endocervix, Vagina, Fallopian tube). Gene expression differences between sexes were examined for each tissue, analyzing a total of 59,033 genes.

A curated subset of 854 drug target genes was retrieved from the ‘druggable proteome’ dataset in the Human Protein Atlas (https://www.proteinatlas.org/humanproteome/tissue/druggable). These genes correspond to targets of FDA-approved drugs and are directly involved in their mechanisms of action, as identified in DrugBank (https://go.drugbank.com/).

*voom-limma* was applied to compare the ${log}_{2}CPM$ gene expression values between Female and Male samples in each tissue^65^. Covariates included age, ischemic time, RNA integrity number (RIN), and surrogate variables (SVs) obtained using smartSVA^66^. Adjusted *P*-values were calculated using the Benjamini–Hochberg procedure to control the False discovery rate (False discovery rate, FDR ≤ 0.05). Additionally, coefficients for sex, age, ischemic time, RNA Integrity Number (RIN), and SVs were used to estimate the effect size of each variable on gene expression.

Subsequently, across-tissue meta-analysis using *MASH* was conducted. For all genes analyzed, *MASH* was supplied with sex effect size and standard error matrices quantified by *voom-limma* to jointly model differential expression across tissues^67^. In contrast to traditional single-tissue analyses or meta-analyses, *MASH* increases power by modeling arbitrary correlations in multi-tissue differential expression. This approach allows the detection of both tissue-specific and shared effects. *MASH* provides estimated effect sizes (posterior means) along with their corresponding standard errors (posterior standard errors) and measures of uncertainty, including Local False Discovery Rate (LFDR) and Local False Sign Rate (LFSR). LFSR was used as a conservative statistical metric compared to LFDR, allowing for the evaluation of the effect’s directionality. Significance was defined as LFSR ≤ 0.05.

Consequently, statistically significant sex-biased genes were identified using the LFSR obtained from *MASH* analysis and the FDR from *voom-limma*. (FDR ≤ 0.05, LFSR ≤ 0.05) and the effect sizes were determined using the posterior means derived from *MASH*. In *voom-limma*, the coefficient is considered as the fold change since it is obtained after converting the gene’s CPM values to ${log}_{2}$. In the *MASH* analysis, the posterior mean was derived using these coefficients, and thus, the posterior mean was regarded as the statistically adjusted ${log}_{2}$ fold change. The posterior mean was calculated based on the criterion that positive values indicate higher expression levels in males, while negative values indicate higher expression levels in females.

## Supplementary Information

Supplementary Information.

## Data Availability

GTEx v10 data was obtained from gtexportal.org. The dataset analyzed in this study is provided in the supplementary file.
